# Supplementary material for: Random forest-based prediction of stroke outcome
Source: Sci Rep. 2021 May 12;11:10071. doi: 10.1038/s41598-021-89434-7 (PMC8115135; doi:10.1038/s41598-021-89434-7)
Supplement: Supplementary file 1 — Supplementary Information [file 41598_2021_89434_MOESM1_ESM.pdf]

Random forest-based prediction of stroke outcome

Carlos Fernandez-Lozano[1,2], Pablo Hervella[3], Virginia Mato-Abad[4], Manuel Rodríguez-Yáñez[5], Sonia Suárez-Garaboa[4], Iria López-Dequidt[5], Ana Estany-Gestal[6], Tomás Sobrino[3], Francisco Campos[3], José Castillo[3], Santiago Rodríguez-Yáñez[4*], Ramón Iglesias-Rey[3*]

[1]Department of Computer Science and Information Technologies, Faculty of Computer Science, CITIC-Research Center of Information and Communication Technologies, Universidade da Coruña, A Coruña, Spain

[2]Grupo de Redes de Neuronas Artificiales y Sistemas Adaptativos. Imagen Médica y Diagnóstico Radiológico (RNASA-IMEDIR). Instituto de Investigación Biomédica de A Coruña (INIBIC). Complexo Hospitalario Universitario de A Coruña (CHUAC), SERGAS. Universidade da Coruña, A Coruña, Spain

[3]Clinical Neurosciences Research Laboratory (LINC), Health Research Institute of Santiago de Compostela (IDIS), Santiago de Compostela, Spain

[4]Software Engineering Laboratory, Department of Computer Science and Information Technologies, Faculty of Computer Science, University of A Coruña, A Coruña, Spain

[5]Stroke Unit, Department of Neurology, Health Research Institute of Santiago de Compostela (IDIS), Hospital Clínico Universitario, Santiago de Compostela, Spain

[6]Unit of Methodology of the Research, Health Research Institute of Santiago de Compostela (IDIS), Santiago de Compostela, Spain

<u>Address for correspondence</u>:

Ramón Iglesias-Rey (ramon.iglesias.rey@sergas.es)*
Santiago Rodríguez-Yáñez (santiago.rodriguez@udc.es)**

*Hospital Clínico Universitario, Rúa Travesa da Choupana, s/n 15706 Santiago de Compostela, Spain. Telephone/ Fax number: +34 981951098/+34 981951098
** Faculty of Computer Science, Campus de Elviña 15071 A Coruña, Spain. Telephone/ Fax number: +34 981167000/+34 981167160

Supplementary Material

Figure S1: We ran several experiments, some of them with baseline models such as KNN). Following the same experimental design (100 runs, 10-fold, hyperparameter tuning and same initial seeds) the results for our initial (using all the available features in the dataset) and final experiments are shown.

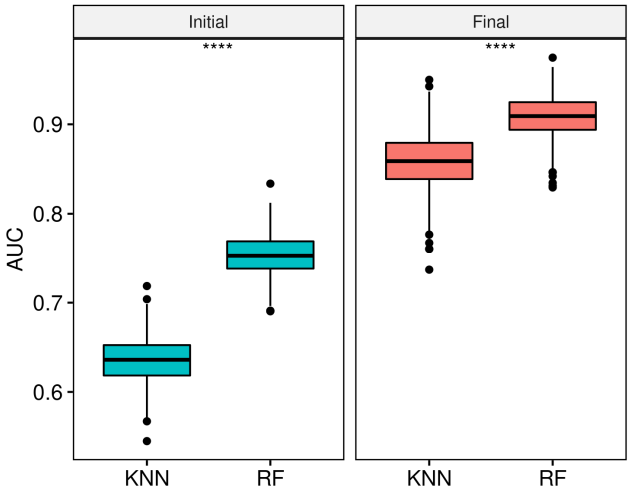

Data pre-processing. We tested with a Shapiro-Wilk (widely recommended for normality test as it provides better power than others such as de Kolmogorov-Smirnos) if our results are normally distributed. From the output, W = 0.92335 (p-value < 2.2e-16), implying that the distribution of the data is significantly different from the normal distribution. In other words, we can not assume the normality and we performed two different Wilcoxon tests, one for the initial experiments and the other with our final approach (feature selection). In both cases, we achieved significant p-values. Thus, according to the results in the initial condition we realized that RF is a good choice and it's statistically better than baseline models. Following our experimental design using feature selection we observed that both baseline models and RF increase significantly its performance (we have noisy and irrelevant features that our filter feature selection detected and removed) and, even more, RF is again statistically better than baseline

models. Finally, we consider that internal model explanation is critical in our analysis so, in this case, RF is a good choice as we can extract an importance score for each one of the selected features.
